# AI-based hardware and software tools in microscopy to boost research in immunology and virology

**DOI:** 10.3389/fimmu.2025.1610345

**Published:** 2025-09-30

**Authors:** Diego Morone, Rocco D’Antuono

**Affiliations:** ^1^ Università della Svizzera italiana (USI), Faculty of Biomedical Sciences, Institute for Research in Biomedicine, Bellinzona, Switzerland; ^2^ Graduate School for Cellular and Biomedical Sciences, University of Bern, Bern, Switzerland; ^3^ Crick Advanced Light Microscopy STP, The Francis Crick Institute, London, United Kingdom; ^4^ Department of Biomedical Engineering, School of Biological Sciences, University of Reading, Reading, United Kingdom

**Keywords:** microscopy, immunology, virology, machine learning, deep learning, image analysis, feedback microscopy, smart microscopy

## Abstract

The integration of computational advances in microscopy has enhanced our ability to visualise immunological events at scales. However, data generated with these techniques is often complex, multi-dimensional, and multi-modal. Data science and artificial intelligence (AI) play a key role in untangling the wealth of information hidden in microscopy data by enhancing image processing, automating image analysis, and assisting in interpreting the results. With this Review, we aim to inform the reader about the advances in the fields of fluorescence and electron microscopy with a focus on their applications to immunology and virology, and the AI approaches to aid image acquisition, analysis, and data interpretation. We also outline the open-source tools for image acquisition and analysis and how these tools can be programmed for an image-informed, AI-assisted acquisition.

## 1 Introduction

Since the first observations of microorganisms using a high-quality single-lens microscope by Antonie van Leeuwenhoek in the 17th century (1), microscopy has been instrumental in understanding diseases and alterations of the immune system. In the late 19th century, Robert Koch isolated the anthrax and tuberculosis bacteria, establishing microscopy as one of the key techniques for investigating the immune system (2). Today, microscopy offers unparalleled insights into molecular mechanisms, cellular dynamics, and tissue interactions.

Before the 20th century, observations were performed using brightfield light microscopy (3). Afterwards, several innovations have led to progress in the imaging of immune cells and molecules. Among them, we mention the optimisation of fluorescent probes in conjunction with the advancement of fluorescence optical methods (4), the development of techniques for antibody isolation (5), the discovery of natural fluorescent proteins (6), and the development of genetic manipulation techniques (7). These innovations allow today the precise imaging of cells and molecules, enabling real-time tracking of dynamics, localisation, and interactions that characterise the immune system both *in vitro* and *in vivo*. On the other hand, the use of electron microscopy (EM) to visualise structures that are relevant for immunology and virology dates back to the first images of viral particles by Ernst Ruska in 1939 (8, 9). Since then, improvements in both protocols and microscope techniques have led to significant increase in resolution, maximum sample size, and tagging specificity. Among these, we should cite progresses in sample preparation, such as ultramicrotomy (10) and cryogenic techniques (11, 12), advancements in microscope hardware, such as the use of digital cameras and direct detectors (13) and scanning electron microscopy (10), or finally advancements in software, for microscope automation (14–17), image reconstruction (18), tomographic reconstructions (19), and alignment between different modalities (20–24). Today, microscopy encompasses a wide range of techniques, ranging spatial scales from the molecular detail to the whole tissue and organ, and time scales from the millisecond to days (25, 26). The wealth of visible and hidden information in the images can deeply enhance our understanding of immune events, if unlocked.

Artificial Intelligence (AI) technologies are pervasive in today’s world and are affecting all fields of knowledge, including science (27). AI enables machines to mimic human intelligence and perform tasks that typically require human cognition, such as learning, problem solving and perception. In microscopy, AI can significantly enhance our understanding by extracting information from images, bridging the gap between scales (28), finding hidden connections within images or between images and other types of data (e.g. genomics or proteomics, 29), and guiding the acquisition in challenging experiments and across modalities (30, 31).

In the first part of this Review, we will survey main microscopy and image analysis techniques, with a focus on their use in immunology and virology. The second part introduces the reader to the concepts of AI and its main applications in microscopy, in particular for immunology and virology research. We finish with a discussion on the current challenges in AI and how its integration with microscopy can drive a new generation of tools to unlock novel insights into the immune system.

## 2 Microscopy and image analysis for immunology and virology

### 2.1 Electron microscopy

#### 2.1.1 Room temperature EM

Classic, Room-Temperature Transmission Electron Microscopy (RT-TEM) relies on chemical fixation, lipid staining with contrast agents based on heavy metals, resin embedding, and cutting in ultrathin sections. This methodology, mainly established in the 1960s (32), is an excellent way to investigate subcellular morphology and ultrastructure (33). A certain degree of three-dimensional information can be acquired by capturing EM images at varying sample tilt angles and performing tomographic reconstructions (electron tomography, ET in short, 34). RT-TEM has been applied to visualise organelle changes in immune cells, such as in the investigation of the role of autophagy of the endoplasmic reticulum during plasma cell differentiation (35), the disposal of damaged mitochondria in migrating neutrophils through mitocytosis (a novel mitochondrial quality control process, 36), or the mechanisms of antigen presentation by major histocompatibility complex (MHC) in antigen-presenting cells (37–40). Another classical visualisation approach relies on the negative staining of small particles, such as protein aggregates, viral particles, or extracellular vesicles, and it is used in clinical diagnostics for virus identification (41).

Immuno-Electron Microscopy (IEM) highlights specific markers on the structure of interest by immunostaining with target-specific antibodies conjugated to colloidal gold particles (42). Another way to achieve specific staining is by expressing a genetically encodable tag that triggers the deposition of a contrasting agent (43, 44), which was used for example for the visualisation of the role of ectocytosis in terminating TCR signalling in cytotoxic T cells (CTLs, 45).

Scanning Electron Microscopy (SEM) employs a focused electron beam that scans the surface of a sample, generating an array of secondary electrons that are detected and converted into an image (46). This results in a high-resolution image of the sample’s surface. To improve contrast, the sample is frequently coated with metals. SEM has been employed with immunogold staining to map the SARS-CoV-2 receptor ACE2 distribution along the motile cilia in respiratory multiciliated cells (47). Also, SEM showed how the porosity of liver sinusoids reduces antigen recognition by effector CD8+ T cells (48) or how intercellular nanotubes enable mitochondrial trafficking from bone marrow stromal cells to CD8+ T cells, to enhance their fitness and antitumor efficacy (49).

Finally, volume Electron Microscopy (vEM) is an emerging group of techniques that offers unprecedented insights into the three-dimensional organisation and dynamics of immune cells, tissues, and molecular complexes. vEM is based on TEM or SEM. TEM-based techniques, like serial section TEM (ssTEM) and serial section ET (ssET), reconstruct volumes by acquiring sequentially ultra-thin sample slices. On the other hand, SEM-based techniques, including array tomography, serial block-face SEM (SBF-SEM) and focused ion beam SEM (FIB-SEM), scan sample surfaces to produce image stacks for 3D reconstruction (26, 50, 51). In immunology, SBF-SEM has been applied to reconstruct T cells (52) and to elucidate how *Candida albicans* exploits transcellular tunnels to invade epithelial cells while evading host immunity (53). FIB-SEM helped in clarifying how G protein subunit Gβ4 negatively regulates phagocytosis by controlling plasma membrane abundance in myeloid cells (54), or was employed to create a 3D reconstruction of CTLs with target cells (45). FIB-SEM was instrumental in reconstructing, with a near-isotropic resolution of 4 nm, whole-cell organelle segmentations, which resulted in the “OpenOrganelle” web repository (55–57). Among others, a notable example is the reconstruction of a CTL interacting with an ovarian cancer cell (56). Moreover, vEM can be combined with advanced labelling techniques like immuno-gold (58) or fluorescent nanoparticles to visualise specific cellular structures or molecular interactions within complex biological samples (59), providing an invaluable information for understanding the spatiotemporal organisation of immune responses at the ultrastructural level.

#### 2.1.2 Cryo-EM and freeze substitution

Cryo-Electron Microscopy (cryo-EM) techniques can currently achieve the sub-nanometre range (60–62). The sample (proteins, cells, or tissues) is first flash-frozen at cryogenic temperature, allowing the creation of a layer of vitreous ice, which fixes the sample while preserving its ultrastructure (11). The vitrified sample can be visualised by different techniques. Microcrystal Electron Diffraction (MicroED) provides structural information from 3D nanocrystals (63). Single Particle Analysis (SPA) reconstructs protein structures without the need for crystallization (61, 62). Cryo-Electron Tomography (cryo-ET) enables the 3D reconstruction by capturing images at varying tilt angles and performing tomographic reconstructions (34) and together with subtomogram averaging can resolve macromolecules (64). Lastly, cryo-Scanning Transmission EM (cryo-STEM) provides a tomographic reconstruction of thick lamellae with quantifiable chemical characterization (65). Cryo-EM techniques have revolutionised structural immunology, enabling the visualisation, in high-resolution, of viral particles (66, 67) or SARS-CoV-2 assembly and egress (68), the TCR complex assembly (69–72), the structural components of antigen processing and presentation (73), the chemokine recognition and the activation of chemokine receptors CCR5, CCR6, CCR2, CCR3 (74), and helped guiding the design of nanoparticles inducing potent neutralising antibody responses (75).

Cryo-Immuno-EM of ultrathin cryo-sections prepared from chemically fixed samples (76) allows the best preservation of protein antigenicity, as it requires chemicals only for fixation (77). When imaging surface proteins, cells can also be labelled with immuno-gold before cryo-fixation and imaged by cryo-ET (78). This approach opens a range of applications for the study of ultrastructural localisation of surface markers, with potential relevance for the field of immunology.

Volume EM at cryogenic conditions can capture 3D morphology in cells at near native state. It can be approached with cryo-ssET (cryo-serial section Electron Tomography) or with cryo-FIB-SEM. For example, cryo-FIB-SEM showed how growth hormone remodels 3D mitochondrial structure in macrophages (79) or the 3D ultrastructure of HIV virological synapses (80).

Finally, in the case of cells and tissues, samples can be plunge-frozen (81, if less than 10-15 μm in thickness) or fixed by high-pressure freezing (82, 83, if between 20-200µm in thickness). After flash-freezing, the sample can be imaged at cryogenic temperature or slowly brought back to room temperature in a chemical fixation buffer, using a so-called freeze-substitution protocols (84). This approach reduces artefacts that could potentially be introduced by toxic chemical agents used as fixatives. Freeze substitution techniques are also employed in light microscopy to reduce fixation artefacts when performing subcellular diffraction-limited or super-resolved imaging (85–89).

#### 2.1.3 Room-temperature CLEM

**Figure 1:**
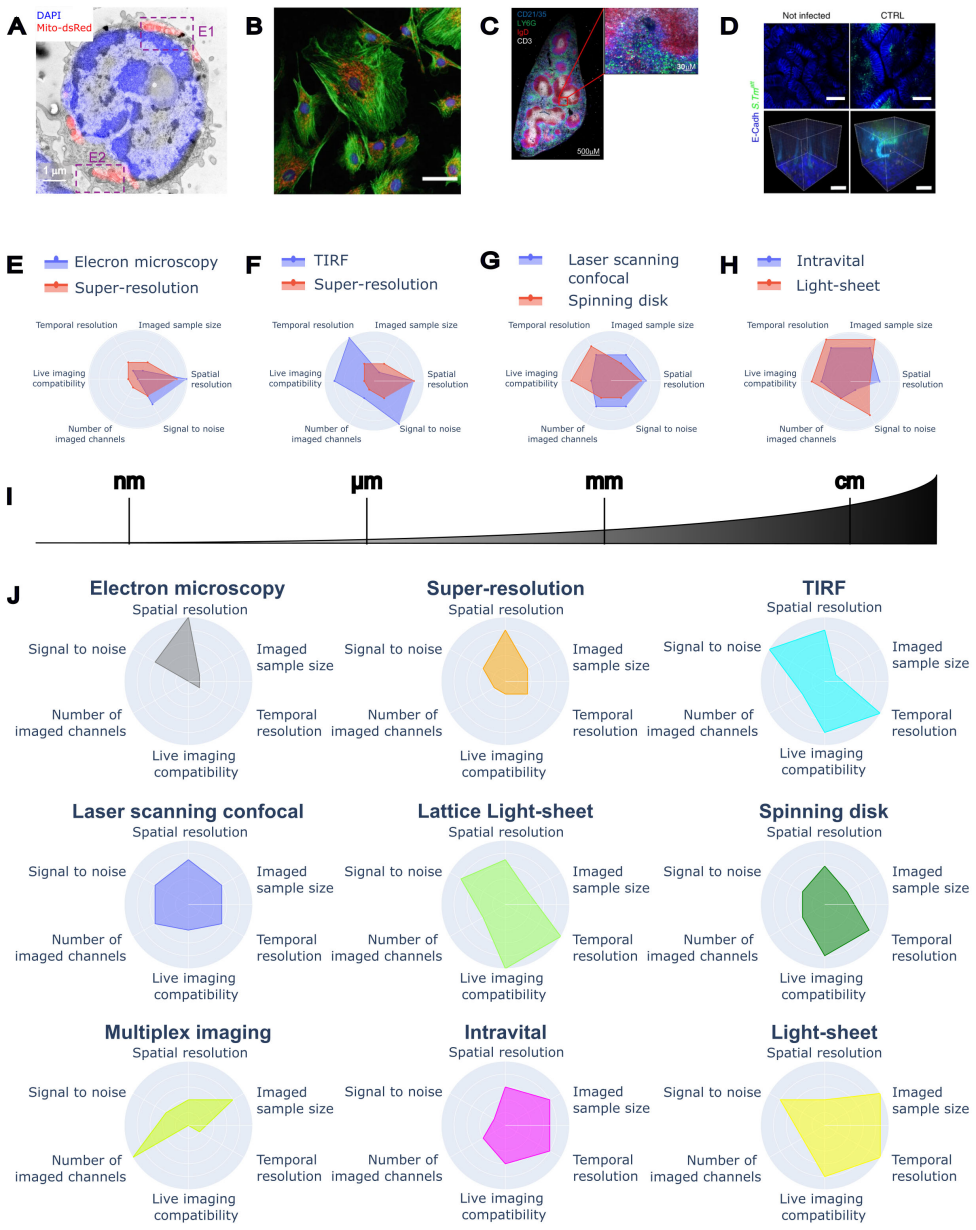
Imaging at scales and performances of different microscopy techniques for immunology. **(A)** Correlative Light-Electron Microscopy of mouse T cell (transmission electron microscopy image in grey, DAPI in blue, and mitochondria in red), reproduced from (49, CC-BY 4.0). **(B)** Fluorescence multichannel image of BPAE cells, reproduced from (90, CC-BY 4.0). **(C)** Whole tissue section of mouse spleen acquired on a confocal microscope, reproduced from (91, CC-BY 4.0). **(D)** Gut bacterial infection with whole crypts imaged in 3D, reproduced from (92, CC-BY 4.0). **(E)** Comparison between Electron Microscopy and Super-Resolution techniques. **(F)** Comparison between TIRF and Super-Resolution techniques. **(G)** Comparison between Laser Scanning confocal and Spinning Disk confocal. **(H)** Comparison between Intravital and Light Sheet. **(I)** The spatial scale covered by different microscopy techniques. **(J)** Radar plots visualising the performances of individual microscopy techniques.

Correlative Light-Electron Microscopy (CLEM) integrates the complementary approaches of light and electron microscopy on the same portion of cell or tissue to overcome the limitations of both techniques, combining the multichannel protein localisation of light microscopy with nanometre resolution of EM (**Figure 1A**). The sample is usually imaged separately with the two modalities and then images are aligned with respect to each other. This poses several challenges to both acquisition and image registration. Some approaches that directly combine both modalities in the same microscope are starting to appear (93). One interesting application of CLEM is the visualisation, in the study by Baldwin et al. (49), of mitochondrial transport from bone marrow stromal cells to CD8+ T cells, by fluorescently labelling mitochondria in stromal cells and then imaging CD8+ T cells with both modalities. Also, FIB-SEM has been combined with light microscopy to visualise the virological synapse and virus-containing compartments in HIV-infected T cells (94). The combination of intravital microscopy and electron microscopy merges the dynamic information of immune cells *in vivo* with a more comprehensive characterisation of the same in fixed tissue. This multiscale deep phenotyping approach is reviewed in (95).

#### 2.1.4 Cryo-CLEM

CLEM combined with cryogenic conditions also has great potential, due to its preservation of the native state, very high resolution and capacity to retain fluorescent signals (96). For example, this approach has been applied to investigate the intracellular trafficking of *Salmonella* bacteria (97). In the case of genetically engineered cells (such as when expressing GFP or other fluorescent proteins), cryo-fixation can then be followed by cryo-sectioning with FIB (98) or cryo-ultramicrotomy (99). Cryo-fluorescence light microscopy (cryo-FLM) can guide the *lamella* milling process (100), also with super-resolution LM (89). Finally, current protocols are extending cryo-CLEM to post-milling visualisation (101), and to 3D samples, such as organoids (102), thus providing a step towards sub-nanometre visualisation in larger 3D context, with relevant applications in immunology.

#### 2.1.5 Conclusions on the use of electron microscopy in immunology and virology

All in all, electron microscopy offers insights into the ultrastructure and three-dimensional organisation of viruses, immune complexes, cells, and tissues that are unattainable with light microscopy techniques (**Figures 1E**, **1J**). Coupled with immunostaining in a correlative approach, EM also informs on the localisation of selected proteins, thus giving context to the ultrastructural detail. EM poses several challenges in terms of image analysis. High-resolution cryo-EM of viral and protein structures employs state-of-the-art algorithms to reconstruct information from low-signal images. Images of thin slices and tomographic reconstructions are frequently analysed by manual segmentation due to the complexity of the ultrastructural contrast. Volume EM reconstructions pose many challenges in terms of image reconstruction, alignment, contrast, and segmentation, due to the size and the complexity of the structures visualised. All these techniques are already or might soon take advantage of the latest AI and image analysis developments (**Table 1**).

**Table 1:** Resolution range, strengths, sample type and AI applications for different microscopy techniques.

Technique	Resolution range	Strengths	Sample suitability	Examples of technique-specific uses of AI
RT-EM	Spatial resolution less than ~1 nm laterally, from less than 10 nm (FIB-SEM) up to 200 nm (ssTEM) axially	High resolution, ultrastructural contrast	Fixed extracellular vesicles, viral particles, cells, organoids or tissues	Segmenting extracellular vesicles with TEM (103), HIV-1 virions with TEM (104) or segmenting α-granules or mitochondria in SARS-CoV2 patient-derived platelets with FIB-SEM (105)
Cryo-EM	Spatial resolution down to atomic resolution	Highest resolution, optimal preservation of sample’s native state	Purified proteins and macromolecules (SPA), pleomorphic objects (virus, vesicles, bacteria) and *in situ* cellular studies without purification and isolation (cryo-ET in combination with cryo-FIB milling and cryo-microsectioning)	Finding macromolecules in cellular 3D cryo-ET tomograms (28), aiding template matching in cryo-ET for particle picking (106)
CLEM and Cryo-CLEM	Depending on the EM and LM techniques used	Multichannel fluorescence specificity and ultrastructural details, also in correlation with live imaging	Live or fixed cells, organoids or tissues	Aiding automated registration (107) and/or segmentation (108)
Super-Resolution	Spatial resolution down to ~1–10 nm laterally, down to 10 nm axially. Temporal resolution down to ~100 ms	Multichannel fluorescence imaging with resolution at the nanometre scale	Depending on the technique: live or fixed molecular imaging in cells, organoids or tissues	In SMLM, increasing acquisition speed (109) and improving resolution (110). In STED, identifying Zika virus reorganization of the endoplasmic reticulum (111)
TIRF	Spatial resolution diffraction-limited (200–200 nm) laterally, down to 80–100 nm axially. Temporal resolution down to ~10 ms	Fast live imaging of dynamic events near cell membrane	Live imaging of purified cell components or cells	Cross-modality transformation from TIRF to SIM (30), improving smFRET tracks analysis (112)
Laser scanning confocal	Spatial resolution from 200–220 nm (diffraction-limited) to the micrometre laterally, from less than 1 to a few micrometres axially. Temporal resolution down to ~100 ms	Flexibility of application, from subcellular detail to live imaging and large tissue reconstruction	Live or fixed cells, organoids and tissues	Resolution enhancement (113), reduction of optical aberrations (114), improvement in FLIM lifetime determination (115)
Lattice Light-Sheet	Isotropic spatial resolution diffraction-limited (200–220 nm). Temporal resolution down to ~10 ms	Ultrafast isotropic reconstruction of small volumes with minimal phototoxicity	Live cells or small organoids	Resolution enhancement and ultrafast super-resolution (116)
Spinning disk	Spatial resolution: same range as Laser Scanning confocal. Temporal resolution down to ~10 ms	Fast 3D imaging of dynamic events	Live cells and tissues	Resolution enhancement and speed improvement (117)
Multiplex imaging	Depending on acquisition technique used (e.g. Widefield or Laser Scanning confocal)	High-content imaging for extended sample phenotyping with multiple fluorescence channels	Fixed tissues	Cell segmentation in complex tissues (118, 119) or detection-based phenotyping (120). Characterization of tumour microenvironment in lung cancer (121) or pancreatic ductal adenocarcinoma (122).
Widefield fluorescence and brightfield imaging	Spatial resolution from 200–220 nm (diffraction-limited) to the micrometre laterally, few micrometres axially. Temporal resolution down to ~10 ms	Fast, low toxicity imaging	Live or fixed cells, organoids and tissue slices	Providing optical sectioning (30, 123, 124) and label-free imaging (125–130). Aiding cell tracking in chemotaxis experiments with brightfield (131)
Intravital	Spatial resolution: same range as Laser Scanning confocal. Temporal resolution down to ~100 ms	4D imaging with high sample penetration	*In vivo* or *ex vivo* tissues and organs	Cell segmentation (132) and resolution improvement in high-speed imaging (133)
Light-sheet microscopy	Spatial resolution in range 1-10 μm. Temporal resolution down to ~10 ms	Fast volumetric reconstruction of large samples with cellular resolution and low phototoxicity	Live or fixed organoids, tissues, embryos, organisms	Resolution enhancement (113, 134), performing segmentation to study nanoparticle delivery to alveolar macrophages (135)

The table summarizes, for each technique, the resolution range, the strengths and uses cases, the applicable sample types and provides examples of application of AI methods for image analysis.

### 2.2 Light microscopy

#### 2.2.1 Super-resolution

By selectively tagging molecules of interest, fluorescence microscopy allows the characterisation of functional and structural features of biological samples, with the intrinsic limitation of the optical resolution limit (136, 137). In fluorescence microscopy, the diffraction-limited resolution is in the order of 200–220 nm, a scale compatible with most cell and tissue imaging applications. However, the scale of many biological structures, such as organelles and molecular clusters, is at least one order of magnitude smaller (tens of nm). Super-resolution microscopy of fluorescent samples bridges the scale of light and electron microscopy, preserving sample integrity and the possibility of performing functional imaging on live samples (**Figures 1E**, **1J**). Most super-resolution methods are based on the manipulation or the analysis of the on/off state of emitters (fluorophores), which are changed either spatially or temporally (138), or on the concept of light reassignment by optical rescanning (139) or pixel reassignment (140).

Localisation microscopy is based on experimental minimisation of the number of active emitters in the field of view, either by activating only a few fluorophores at the time or by limiting the population in the ground energetic state. This allows the determination of the emitter position with the highest probability (141). Localisation microscopy, including techniques such as PALM and STORM, requires the acquisition of a high number of frames to accumulate information about biological structures, and the use of blinking fluorophores to obtain a sparse presence of emitters in the field of view. In general, the definition of biological structures in the final image improves with the number of accumulated frames (in the order of thousands). However, there are methods to optimise the acquisition parameters, such as excitation power and number of frames needed, depending on the structure dimensionality (142), or to identify artefacts in the super-resolved image based on a local error map (143). In immunology, localisation microscopy provided information on SARS-Cov-2 entry in liver spheroids (144), showed how the TCR is randomly distributed on the surface of resting antigen-experienced T cells (145), and informed on the structure of cluster in the NK cell’s immune synapse (146).

One of the methods to achieve super-resolution using specific illumination patterns is the STimulated Emission Depletion (STED) microscopy, in which the illumination laser, hitting the sample as a diffraction-limited laser spot, is used together with a depletion laser illumination, shaped as a toroidal pattern that switches off fluorescence, leaving a smaller emission spot and therefore increasing the resolution as a function of the power of the depletion laser (147). In immunology, STED microscopy has been used to show the role of SWAP70 in organising actin cytoskeleton during phagocytosis (148) and how TIGIT receptor can inhibit T cell activation by forming nanoclusters (149).

Other optical super-resolution methods implemented on laser-scanning systems, such as Zeiss Airyscan (150) and Nikon NSPARC (151), rely on light reassignment, assuming that higher order rings of Airy pattern can be detected with arrayed detectors and light be reassigned to the centre of the pattern where single emitters should be located (140). Finally, other methods such as iSIM and SoRa, are based on optically rescanning the point spread function to reduce its size and obtain instant super-resolution imaging on camera-based systems (152).

Instead, super-resolution methods based on the temporal analysis of fluorescence intensity fluctuations do not require blinking fluorophores and can be employed on data sets acquired on conventional microscopes (e.g. wide-field, TIRF, laser scanning confocal). They are referred to as Fluorescence-Fluctuation based Super Resolution Methods (FF-SRM), and each relies on a different statistical analysis of the temporal fluorescence fluctuations (e.g. Super-Resolution Optical Fluctuation Imaging (SOFI, 153), Super-Resolution Radial Fluctuations (SRRF, 154) or Mean-Shift Super-Resolution (MSSR, 155) to overcome specific limitations in the acquisition or in the image, such as low signal-to-noise, low number of frames, capability to reconstruct hollow structures or susceptibility to the creation of image artefacts (156).

All in all, the landscape of super-resolution microscopy ranges from methods based on light reassignment, providing moderate optical resolution increase (e.g. Airyscan), to methods based on localisation microscopy and fluorescence depletion, achieving a resolution of the order of the nanometre (e.g. RESI (157), MINFLUX (158–160) and MINSTED (161)). The use of computational methods on top of super-resolved images can further enhance super-resolution even with a limited number of frames (155).

#### 2.2.2 TIRF

Total Internal Reflection Fluorescence (TIRF) microscopy uses the total internal reflection of a laser beam to create a thin illumination layer. This allows the observation of fluorescent molecules close to the coverslip surface (depth of about 100–200 nm, **Figures 1F**, **1J** 162, 163). Such method provides high-resolution images of the basal cell layer with minimal background noise, making it ideal for studying cellular processes such as migration, adhesion, and signalling (164).

In immunology, TIRF microscopy is commonly used to study the interactions between immune cells in antigen presentation. For example, seminal studies employed this technique to investigate TCR clusterization and activation pathways following antigen recognition (165, 166). More recently, TIRF has been used to highlight that clathrin is recruited in microclusters to mediate internalisation and vesicular release of a triggered T cell receptor at the immunological synapse (167). Another application of TIRF microscopy in immunology is the study of receptor clustering, such as to show the importance of CD4+ T cell’s CXCR4 nanoclusters in supporting CXCL12-mediated responses (168). On macrophages, TIRF has been employed to show the accumulation of dynamin-2 at the site of phagosome closure (169).

A recent evolution of TIRF microscopy, called quantitative dynamic footprint (qDF) and based on variable-angle TIRF, was used to visualise leukocytes rolling, adhering, and spreading with nanometre-scale z-resolution (170, 171). Finally, TIRF in conjunction with SIM super-resolution microscopy showed the engagement of two spatially distinct TCR microclusters with ZAP70-bound TCR and LAT-associated signalling complex (172).

#### 2.2.3 Confocal

Confocal microscopy is based on the use of an optical aperture, called a pinhole, to obtain the optical sectioning of the sample and localise the fluorescent signal in 3D (**Figure 1B**). It was invented by Marvin Minsky (173) – a computer scientist who would later play a significant role in the development of AI concepts and methods – and has been improved with the use of lasers and scanning systems (174, 175). It avoids the need to physically slice thick samples by rejecting out-of-focus light proportionally to the reduction of the pinhole aperture (176).

Thanks to its versatility, confocal microscopy can find applications on a wide range of samples, from fast visualisation of live subcellular events to reconstruction of large portions of thick tissues. On the subcellular scale, confocal live-cell microscopy was used to investigate how lymphocytes, in the absence of chemotactic signalling, orient their migration against a fluid flow (177), to characterise the force dynamics in phagocytic engulfment by cytotoxic T cells (178), and to show how the Golgi complex directs the positioning of lytic granules inside NK cells to guide their cytotoxicity (179). At the cellular level, tracking of macrophages in live-cell confocal imaging helped, together with modelling, in clarifying how these cells use a collective quorum licensing to initiate inflammation (180). In fixed tissue samples (**Figure 1C**), confocal microscopy has been employed to visualise macrophages in meningeal compartments of the central nervous system (181), vascular endothelium of mouse lymph node (182), virion transport to lymph nodes (183) and neutrophil accumulation (91). Moreover, it helped in defining the role of scavenging chemokines in marginal B cell zone formation (184) or the contribution of innate lymphoid cells and conventional T cells on shaping gut microbiota and lipid metabolism (185), and Treg accumulation around self-activated T cells in lymph node paracortex (186). Finally, it contributed to characterising megakaryocytes in the bone marrow niche (187), periarteriolar alignment and integrin-dependent network formation of tissue-resident mast cells (188), platelets around metastatic niches in lungs (189), and tissue-resident memory T cells on the ocular surface (190). In a high-throughput manner, confocal microscopy was instrumental in isolating CAR-T cell clones with a multi-killing property against patient-derived cancer cell organoids and associating this information with their transcriptomic profile (191).

When coupled with pulsed lasers, confocal microscopy can detect fluorescence lifetimes in so-called Fluorescence Lifetime Imaging (FLIM, 192). The determination of the lifetimes can be done either with exponential fit of the decay histogram or with phasor analysis (193). The measured lifetimes are concentration-independent but microenvironment-dependent. Thus, local microenvironment changes can be assessed, such as pH and ion changes, FRET events (194) or cell membrane tension (195). Confocal microscopy can also be used to visualise structures below the diffraction limit by means of expansion microscopy, which increases the sample size (e.g. by 10-fold), and standard confocal imaging (196). Finally, confocal live acquisitions can offer insights into molecular behaviour with techniques such as Fluorescence Correlation Spectroscopy (FCS, 197), Image Correlation Spectroscopy (ICS, 198), and Number and Brightness (N&B, 199). For example, N&B has been applied to determine GPCR oligomerisation states in live cells (200).

Recently, new technologies in confocal imaging are being developed to increase its speed and multi-view capabilities, such as techniques for fast, super-resolution, and multi-view imaging (201) or virtual scanning light-field technologies (202, 203).

#### 2.2.4 Confocal and multiphoton for intravital imaging

Staying true to the microscopists’ motto, “Seeing is believing” (204), intravital microscopy (IVM) addresses the need to observe events in their context (205), which provides complementary information to static 3D tissue phenotyping (206). Depending on the degree of tissue transparency and the required depth of imaging, IVM can be achieved with widefield, confocal, or multiphoton microscopy. Due to the shallow imaging capabilities of widefield microscopy, most studies are conducted using confocal or multiphoton approaches.

In the case of confocal, IVM is sometimes based on the use of a faster alternative microscope called Spinning Disk (SD, **Figures 1G**, **1J** 207). Here, multiple excitation points are obtained by splitting the laser beam with microlenses on a rotating disk, while corresponding pinholes on a second rotating disk perform optical sectioning. Spinning disk microscopy has been applied to visualise Kupfer cells sequestering *E. coli* to show how this mitigates neonatal sepsis (208), liver-specific Treg and their re-programming of liver neutrophils (209), peritoneal macrophages (210), patrolling by alveolar macrophages (211), and mechanisms of control of dendritic cells by nociceptors (212). A promising approach to confocal IVM is the recent development of confocal light-field microscopy (203, 213), which achieves real-time acquisition of whole volumes (Z-stacks) with micrometre resolution.

Multiphoton microscopy (MPM) combines laser scanning with a multiphoton near-infrared excitation. It is based on the simultaneous absorption of multiple low-energy photons, resulting in the same fluorescence emission as in conventional one-photon excitation. Multiphoton excitation increases the achievable imaging depth (**Figure 1D**) thanks to the use of near-infrared wavelengths that are scattered less by the sample, eliminates out-of-focus excitation and reduces phototoxicity and photobleaching (214). MPM contributed to several major discoveries in immunology (see reviews 205, 215). The most used type of multiphoton excitation is by means of two photons (also called Two-Photon Microscopy, TPM). Examples of application of TPM in immunology include the visualisation of inflammatory dendritic cells (216–218) and neutrophil efferocytosis (219) in trachea after influenza infection, innate immune responses in the skin during wound repair (220, 221), macrophage aggregation in a peritoneal sterile wound model (222). Moreover, it helped in investigating chemotactic neutrophil migration bias at capillary bifurcations (223), and complement activation in draining lymph nodes following dermal infection (224). In adaptive immunity, TPM contributed to the show that T cell activation occurs in three stages (225), or to elucidate T cell regulation by innate lymphoid cells in the liver (226), corneal tissue-resident T cells localising at the surface of immune privileged eye (190), mechanism of additive cytotoxicity by CTLs (227), dynamic interaction between marginal zone B cells and red blood cells (228), and B cell control of affinity by restraining somatic hypermutation through controlled cell proliferation (229). TPM also aided *ex vivo* imaging, such as in the visualisation of the role of ATP in limiting protective IgA against enteropathogens (92), or in the visualisation of collagen deposition and mesothelial cell activation in the intraperitoneal gut following microbial contamination (230).

Three- and four-photon excitations have been instrumental to reconstruct the entire depth of a popliteal lymph node (231) or the deep vasculature in brain tumours (232), to the quantification of calcium events in astrocytes in deep portions of tissue (233), and to the acquisition of multichannel data sets (up to 6 channels) in tumour tissues (234). The combined use of TPM and FLIM imaging allows the characterisation of pH and metabolic changes *in vivo* (235). Finally, to overcome the speed limitations inherent to laser scanning systems, faster implementations have been developed that use a synthetic aperture microscopy to achieve long-term imaging at high speed (236) or with a scan-less multiphoton setup for fast, deep, imaging-based neuron voltage recordings (237). On the other hand, adaptive optics methods have been employed to limit scattering in deep tissues and correct aberrations (233, 238). Finally, sample drift or organ movements can pose challenges that AI could address during or after acquisition.

#### 2.2.5 Multiplex imaging

The goal of understanding biological function within the complex context of tissues – which is of particular importance in immunology – led to the development of techniques for visualising and analysing multiple targets or markers within the same sample. Standard imaging setups are usually limited to very few markers at the same time, while multiplex imaging extends the total number of markers in the order of several tens (239).

Current approaches to multiplex imaging include fluorescence imaging, imaging mass spectrometry, or sequencing techniques. In its fluorescence declination, samples are either stained with many fluorophores simultaneously or repetitively stained with fewer fluorophores in many cycles of imaging and fluorescence bleaching (240). Samples are then acquired in widefield or confocal microscopy, to achieve a cellular resolution (**Figure 1J**). When combining multiple fluorophores at the same time, several techniques have been developed to ensure the separation of highly overlapping emission spectra, either based on hardware, such as the employment multiple emission windows and spectral unmixing algorithms (241, 242), or by calculating spill-over with single-stain samples (239). In the case of cyclic imaging, usually two or three fluorophores are used per cycle, with repetitive staining, imaging and bleaching phases, as applied to fixed tissues (243–245), cells (246) and live samples (247). Multiplex imaging techniques require a solid antibody validation, which is addressed also by community efforts (248). Cycling imaging is time-consuming, then a possible improvement is the use of fluorescent tags with DNA barcoding: the sample is stained simultaneously with antibodies tagged with orthogonal single-stranded DNA sequences and then imaged in cycles by using an eraser strand between each cycle (249). Finally, recent and promising advances in fluorescent multiplex imaging use FLIM to increase the number of detectable fluorophores or discriminate the autofluorescence contribution (250). In this regard, techniques using AI to overcome the limitations of low photon budget when performing spectral FLIM imaging are of particular interest (115, 251).

Mass-spectrometry based techniques employ a raster-scanned ionising beam to analyse a small portion of the sample that is then associated with a single pixel in the resulting image reconstruction. Material collected can be endogenous, such as proteins, metabolites, lipids, or glycans (252), or exogenous, as in the case of antibody staining with tags suitable for mass spectrometry, such as peptides or rare metal elements. For example, Imaging Mass Cytometry (IMC) and Multiplexed Ion Beam Imaging (MIBI) can reach single-cell resolution, allowing highly multiplexed spatial proteomics (253, 254). With lower resolution (clusters of cells), metabolite mapping has been performed with both mass spectrometry and Raman spectro-microscopy (255). Overall, the development of these techniques greatly increased the amount and quality of data extracted from samples. Furthermore, the mentioned techniques can be combined in a multi-omics approach to increase sample information (256–258). Of interest are methods that couple automated laser microdissection with shotgun lipidomics (259) or with (epi)genomics and transcriptomics, as they integrate imaging, analysis, and hardware feedback steps to extract interesting information for subsequent analysis (260).

Data obtained with multiplex imaging techniques can pose numerous problems regarding analysis due to size, complexity, and heterogeneity. For example, in the case of fluorescence imaging, removing autofluorescence from paraffin-embedded tissues or complex tumour tissues can be hard to achieve (261). Other challenges in the image analysis include segmentation of cells in the complex tissue environment (240), and spectral separation in the case of one-shot imaging with many overlapping fluorophore spectra (262). On the data interpretation side, data clustering and dimensionality reduction are needed to navigate complex multi-channel data sets and integrate these data with other multi-omics approaches (263). An example is the integration of multiplex imaging and spatial transcriptomics to follow thymic evolution (264). Finally, sharing code and protocols of the analysis pipeline ensures a dissemination of techniques and best practices, fostering the improvement of data analysis pipelines (118). All these tasks can be approached with standard or AI-assisted image analysis techniques, as discussed in the second part of this Review (see Section 3).

#### 2.2.6 Light-sheet microscopy

Light-Sheet Fluorescence Microscopy (LSFM, also called Selective Plane Illumination Microscopy, SPIM) achieves 3D sectioning by illuminating the sample with a thin sheet of light and collecting fluorescence emission in a plane orthogonal to the illumination (265). A volume reconstruction can be obtained by translating the beam or the sample in a single direction or by rotating the sample to perform a tomographic reconstruction. This type of illumination achieves a very fast volume reconstruction with micrometre resolution (**Figures 1H**, **1J**), high signal, low photobleaching and phototoxicity (266). In live samples, this technique has been applied to high-throughput live imaging of T cell cytotoxic function against B-cell lymphoma or the interaction of Tregs with gastric tumour spheroids (267).

A notable application of LSFM microscopy in immunology is the reconstruction of fixed, cleared organoids and tissues. Clearing removes the unwanted tissue components and improves the uniformity of tissue refractive index, thus reducing light scattering and improving image quality at high depth (268). LSFM has been applied to reconstruct many organoid types (269). Its use on cleared tissues is particularly interesting for the whole-organ characterisation of immune landscape and vascularisation of the brain (270), clinical identification of melanoma metastasis in the human lymph node (271), and whole-mouse cleared tissue imaging (272–275).

Lattice light-sheet can image subcellular details with an illumination pattern that achieves diffraction-limited isotropic resolution and high acquisition speed (276). This technique was applied, for example, to the study of the interaction between tumour-associated macrophages and CD8+ T cells (277) or to characterise the effect of antigen strength on immune synapses (31). Given the lower penetration depth of visible light compared to multiphoton illumination, the application of LSFM *in vivo* has been limited to investigating cleared samples, as with the organoids mentioned above, or in embryo development studies (272, 278). However, the development of multiphoton light-sheet systems (279), or the recent implementation of the NIR-II illumination (1000–1700 nm) to light-sheet microscopy opened a window to the feasibility of deep tissue LSFM *in vivo* (280). On the other hand, a variation of visible-light LSFM called Swept Confocally Aligned Planar Excitation (SCAPE) microscopy (281), was applied to the histopathological characterisation of live tissues from their autofluorescence (282). SCAPE provides information on tissue architecture with cellular resolution, with a strong potential for diagnostic applications.

Overall, LSFM is an exciting field, but numerous challenges remain to be addressed. For instance, the vast amount of generated data renders archiving, pre-processing, visualisation, and analysis considerably more complex than other microscopy techniques (283). Many platforms have been developed to tackle the analysis of these complex and big data sets (284). As outlined in a recent review by Daetwyler and Fiolka (266), we also foresee that light-sheet microscopy, with its fast-imaging capabilities, 3D reconstruction of big volumes and generation of highly informative data sets, will take a central stage in microscopy to image cell-cell interactions in complex 3D structures such as organoids and tissues. Also, the resulting data sets are already pushing the generation of novel data analysis techniques (285, 286).

#### 2.2.7 Conclusions on the use of light microscopy for immunology

In conclusion, light microscopy techniques can span resolutions from the nanometre to the centimetre (**Figure 1I**). They significantly advanced our understanding of the immune system, by enabling researchers to visualise complex subcellular structures, cellular interactions, and dynamic processes, for immune phenotyping and dynamic live analyses. Yet, biology occurs across all spatial and temporal scales, while current techniques can only see a portion of these events (25). To cover these different scales, we need both progress in imaging techniques, as well as automated analyses that can inform and guide the capture of events across scales in real-time.

### 2.3 Image analysis

Image analysis is a crucial component of microscopy research, enabling the extraction of quantitative data from complex visual data sets in an unbiased manner (287). The standard toolbox for image analysis comprises tools for image preprocessing, segmentation, tracking, and quantification. Automating this process ensures unbiased data analysis and simplifies compliance with good practices for data and image acquisition reporting (288).

Image preprocessing seeks to minimise noise, enhance contrast, correct geometric distortions, and improve resolution, thus facilitating the subsequent image analysis steps. Denoising techniques improve the signal-to-noise ratio (289): methods vary from standard Gaussian blur and median filter to more advanced techniques such as 3D block-matching (290), non-local means (291, 292), and wavelet transforms (293). Improvements in contrast and resolution can be achieved with deconvolution techniques, where the information about the point spread function of the microscope is used to remove the signal contribution from out-of-focus planes and surrounding signal sources (294). Resolution and contrast improvements can also be obtained by acquiring the same image with slight changes in the illumination beam (295, 296), with general algorithms considering the noise distribution (150, 151), analysing fluorescence fluctuations with algorithms such as mean shift vector analysis (MSSR, 155), or by deconvolution, like in SUPPOSe (297) or B-SIM and Sparse-SIM for SIM images (298, 299). Image registration corrects image distortions and time drift: this is especially useful when reconstructing a volume in a mosaic (300–302), aligning images in case of sample drift, as needed in intravital microscopy (303–306) or aligning images acquired with different modalities, such as in the case of CLEM (51). Lastly, crosstalk correction improves channel separation, eliminating unwanted spectral bleed-through between channels (4, 174), while spectral unmixing techniques use the characterisation of the emission profile to separate many fluorophores with overlapping emission spectra (241). These approaches are particularly useful in multiplex imaging (239) and when subtracting unwanted autofluorescence contributions (242). Other methods for spectral separation include phasor analysis based on fluorescence spectral data, or on fluorescence lifetime (307). Overall, effective image preprocessing greatly simplifies image segmentation, ultimately improving the extraction of quantitative data from microscopy images.

Segmentation of image data is the process of separating the pixels of the background from the pixels of interest, labelling the objects (e.g. organelles, cells, tissue areas) so that properties such as geometrical descriptors can be measured, or intensity statistics be calculated. Image segmentation is at the basis of most automated analysis workflows (308). Segmentation techniques can be broadly categorised into two main groups: region-based and edge-based methods. Region-based segmentation algorithms group together pixels with similar attributes (e.g., intensity, colour) to form homogeneous regions within an image. These algorithms often use intensity thresholding (309), clustering (310), or watershed (311) to identify meaningful segments. On the other hand, edge-based segmentation focuses on identifying boundaries between objects in an image by detecting abrupt changes in pixel attributes, such as intensity or texture. Common edge detection methods include Canny (312, 313), Sobel (314), and Laplacian of Gaussian (LoG) operators (308). The choice of segmentation technique may vary depending on the specific application, where factors to consider include image complexity, object shape, size, and contrast with respect to the background. Object segmentation can then be followed by object classification according to some measurable property, such as object position or shape factors.

Object detection localises objects or regions of interest in an image. The task typically involves identifying the object to be detected (classification) and determining its position in the image (localisation). Object detection is frequently employed to recognise areas of interest, such as a compartment in a cell or tissue (315), and to identify cells in time-lapse microscopy movies for object tracking (316).

Tracking allows the study of temporal dynamics, such as cellular or subcellular movements. Tracking objects in a movie is a two-step process, where object detection and segmentation are followed by object linking between frames (317, 318). Manual or semi-automated tracking software has dominated the scene in studies of immune cell mobility upon antigen presentation, in cell culture experiments, or in lymph node imaging (319). However, when temporal sampling cannot be done at high frequencies, or the linking process is ambiguous, deep learning techniques may prove helpful in enhancing the effectiveness of classical methods (316, 320).

Recent advances in bioimage analysis are significantly broadening its accessibility, allowing researchers to leverage powerful techniques with reduced reliance on programming expertise and lowered computational resource demands. These developments are driven by a growing trend toward simplified user interfaces (321–326), and standardised analysis protocols for light microscopy (327) and electron microscopy (57). However, while these tools streamline many routine analyses, complex or novel research questions often require more sophisticated approaches. This is where the role of the bioimage analyst remains crucial – bridging the gap between readily available software and advanced techniques, and facilitating the development of custom solutions to address unique research challenges (328).

### 2.4 Impact of open-source software and open hardware in image acquisition

Open-Source software (OSS) has a key role in bioimage analysis because the access to source code enables any researcher to develop customised workflows, even with a limited knowledge of programming languages, thus guaranteeing more transparency and reproducibility (329).

Image quantification has been made easy in the past decades by many graphical user interfaces (GUI) suites, among which the most renowned include ImageJ or FIJI (330), CellProfiler (323), napari (331), QuPath (332). The key aspect that makes these GUIs widely adopted is the abundance of scripts and plugins, together with the possibility to access the source code and develop custom bioimage analysis solutions, whether implemented as point and click interaction or as a script.

A fundamental role of facilitator for bioimage analysis based on scripting has been covered by development environments such as RStudio^1^ (based on R language), JupyterLab (333, based on Python) or visual programming suite KNIME (334). Because of the richness of the Python environment, in terms of availability of packages, many bioimage analysis solutions have been developed as Jupyter notebooks, especially in the context of machine learning (ML) and deep learning (DL), where code modification might enhance the adaptation to specific image data (118, 335, 336). The availability of complete notebooks where all the steps of a workflow are explained with code comments facilitates the execution by the end user, learning, and reproducibility (335).

The integration between the full control of microscope motorisation (337), image acquisition, real-time (or offline) image analysis, and the possibility to drive a new image acquisition, based on the result of the analysis, constitutes the backbone of what is called feedback microscopy (338), also referred to as smart microscopy (339). The purpose of such integration is to allow the adaptive imaging of the biological sample in the spatial and temporal dimensions (340).

**Figure 2:**
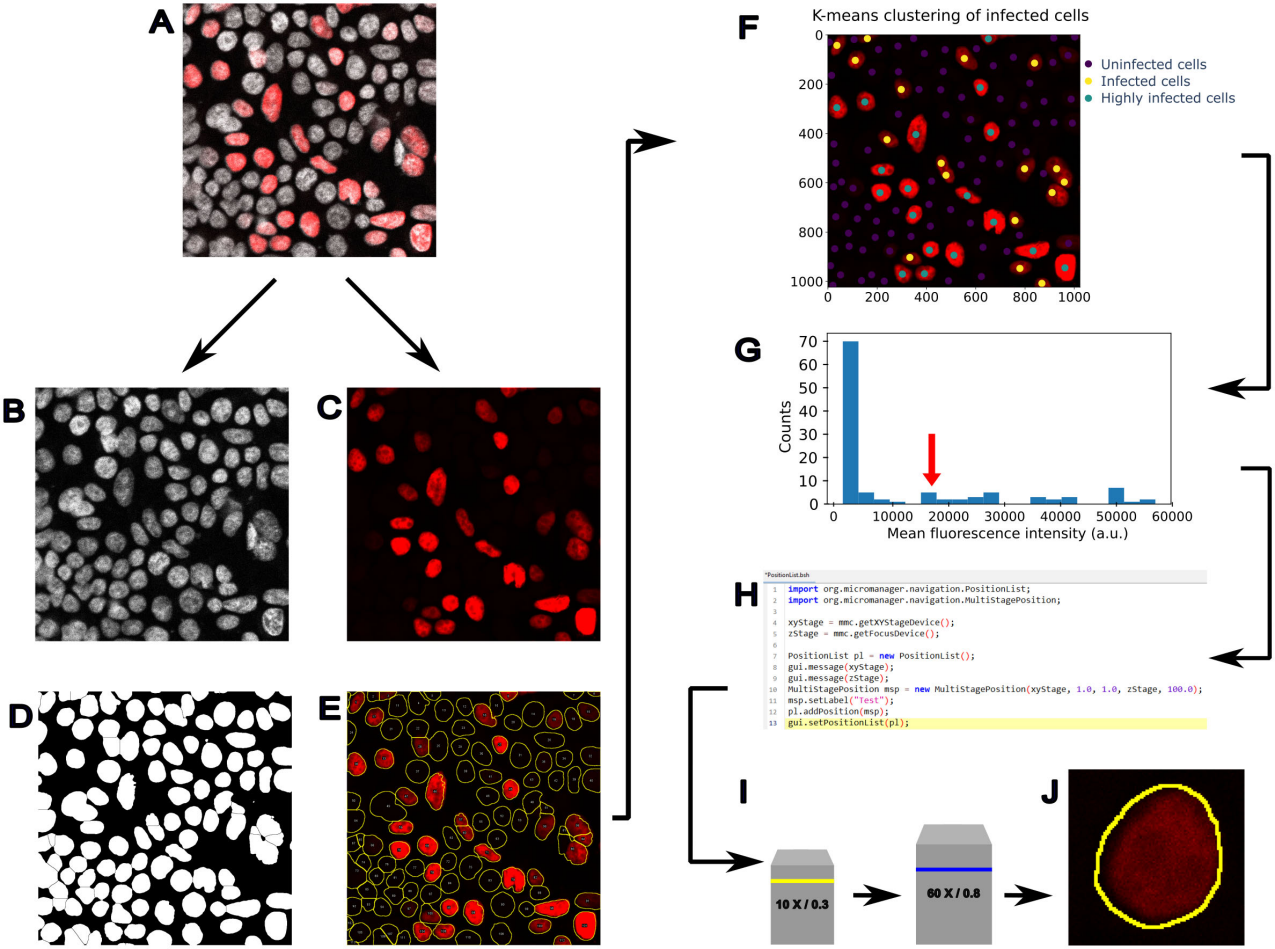
Example of feedback microscopy based on machine learning to analyse infected cells. An application of feedback microscopy to detect infected cells and trigger the acquisition at higher resolution. **(A)** Fluorescence image showing a nuclear marker (grey) and infection reporter (red). **(B)** The nuclear marker channel is used to identify all the cells, while **(C)** the infection reporter is used to detect the infected cells. **(D)** Binary mask obtained by thresholding the nuclear marker image in **(B, E)** The segmentation outlines are used to measure the fluorescence in the second channel (red) and assess the level of the infection reporter. **(F)** K-means clustering can be used to identify cell populations and drive the acquisition of infected cells at higher resolution. In this image, the k-means clustering is initialised by assuming 3 cell populations corresponding to possible levels of infection. After clustering, the cell centroids are labelled with coloured dots, according to the level of infection: uninfected (dark blue dots), infected cells (yellow dots), and highly infected (green dots). **(G)** The infected cells classified by the k-means clustering show a higher level of fluorescence, and cells with a specific value of fluorescence reporter can be identified to trigger a **(H)** repositioning of the stage at a specific location (X,Y), executed through a script (e.g. Micro-Manager beanshell script) and a **(I)** change of objective lens. Finally, **(J)** the identified cell can be acquired at higher resolution.

In **Figure 2**, we present a possible workflow of feedback-based microscopy to identify infected cells and acquire them at higher resolution, minimising the overall acquisition time. A multichannel fluorescence image, including a nuclear marker and an infection reporter (**Figures 2A–C**), is used to identify all the cells in the field of view (**Figure 2D**) and measure the level of an infection reporter (**Figure 2E**). Using a ML clustering algorithm (e.g. k-means clustering), cells are classified based on their infection state (**Figure 2F**). Then, cells are selected depending on their level of infection (**Figure 2G**), and the microscope is instructed to navigate to the cell position (**Figure 2H**), switch objective (**Figure 2I**), and acquire a higher-resolution image (**Figure 2J**). This type of workflow presents the advantage of scanning larger areas to increase the number of inspected cells and use higher resolution imaging only for the infected ones, which are identified with an unsupervised ML algorithm.

Examples of software tools for feedback-based microscopy include AutoscanJ, for the detection of mitotic cells or chromosomal anomalies, based on the same principle of rescanning cells of interest, previously detected with lower magnification (341) or the real-time drift correction in intravital movies (342). In the field of RT-EM, a similar approach has been developed by SerialEM software (14) and its Python interface pyEM (343). This approach was used in the contests of immunological research to show, with tomographic reconstructions, that plasma cells in patients with multiple myeloma display elongated centrioles (344). Another application has been developed for combining light microscopy and FIB-SEM (336). In single-particle cryo-EM, automatic acquisition is even more important because thousands of images are needed to perform a structural reconstruction (17, 345). In cryo-ET, machine learning approaches were used to fully automate *in-situ* cryo-ET workflow (346).

While some scripting tools, such as ImageJ macro language, are very popular in the bioimage analysis community (347), the complexity of back-end programming languages (e.g. C or Java) to develop software plugins may hinder the quick implementation of novel ideas. To facilitate the use of feedback microscopy, projects like Pycro-Manager (348) or pymmcore^2^ have been created to implement translation layers between programming languages (in this case, Python can be used to write scripts rather than Java). On the other hand, the Open-Source Hardware (OSH) movement has allowed the implementation of cheaper solutions for image acquisition compared to proprietary microscopy software. Projects like Micro-Manager (349) to control microscope hardware have revolutionised the field, decoupling the need for commercial licences to operate devices from the mere possession of the equipment. In addition to gaining control of microscopy equipment, the possibility to trigger and modulate the image acquisition with plug-in electronics, for example based on Arduino^3^ or Raspberry Pi^4^ development boards, has widened the possibilities to customise every microscopy platform. However, technology development requires the developers or early adopters to carry the risks of investing resources in technologies that might have limited or delayed benefits. Then, the advantage has to be identified either in the reduced cost of existing open technology or in access to bleeding-edge techniques, which might be rewarded in terms of scientific publications (350).

Finally, Computer-Aided Design (CAD) for machining or 3D printing of microscope components or auxiliary devices has improved the use of resources to run microscopy experiments. Examples include the open optical setup of light-sheet system openSPIM (351), the possibility of fully 3D printing experimental tools or the wide database of open hardware projects developed by the imaging community (for example, by the LIBRE hub^5^). These can include accessories such as syringe injection motors, sample supports, frames for optical filters, enabling components based on electronics (352), or even part of the microscope body, etc. (353). The adoption of OSS or OSH solutions is also strictly dependent on their discoverability, modularity, and the standardisation of the software interface (354).

The revolution of openness in scientific software and hardware is not necessarily in opposition to the business model of microscopy companies (355). In fact, at the request of bioimaging researchers, many companies offer support for the use of open software, such as OMERO (supported by Glencoe^6^ 356), or have opened part of the software by offering an API to interact with some of the GUI modules, such as Zeiss^7^ with APEER platform for deep learning (357) or Abberior^8^ with the possibility to reprogram the hardware configuration (358). In addition to the highly beneficial effect on the broader research community, we believe that companies can also benefit from the openness of both software and hardware. This applies whether resources for science are scarce or research is well funded, because there is always a business model that can be adapted to provide a service for less experienced users (355), and the wide adoption of open imaging solutions by companies enlarges potential customer markets.

The need for openness is even more pressing when AI solutions are implemented, as AI methods are inherently based on probability and as such not prone to reproducibility, and often the general audience employs such methods without a thorough understanding of their applicability and limitations. The second part of this Review aims to clarify some of the concepts and uses of AI for microscopy and immunology.

## 3 Artificial intelligence for microscopy with applications in immunology and virology

### 3.1 Historical introduction to AI

The concept of artificial intelligence takes root in Leibniz’s *characteristica universalis*, a common unified language of pure thought in which every language could be translated, and in *calculus ratiocinator*, a machine capable of replicating that language (359). Computers were meant to take a set of rules and input data and return some output, but could they think autonomously or even generate novel ideas (360)? This question is still guiding research in the AI field. In the 1940s and 1950s, progress in computational capacity motivated people to explore applications in the domain of pattern recognition, where the human brain excelled. In a seminal paper, McCulloch and Pitts (361) proposed the model of a network that took inspiration from the structure of the brain. This network was composed of a single input and output neuron, with an activation state that would contribute to the final output of the network. Later, developments on this original idea extended network complexity to a multi-layer network (362) and developed algorithms to train networks with more than one layer (backpropagation, 363–365).

In today’s technologies, artificial Neural Networks (NN) are built from a collection of nodes (neurons), operating a set of transformations on the input data to learn different representations of it. Nodes can be aggregated in layers and are connected by activation functions (synapses) computing a weighted sum of their input data. When the NN is trained, some connections get stronger, causing them to acquire a higher weight, while others get weaker, thus reducing their weight. So, NN training is essentially a problem of optimisation of parameters (366, 367). Training occurs iteratively: at each cycle, the network’s weights are optimised, and a loss function — an objective measure of training success — is measured (368). This process continues until a stopping point, such as reaching a specified value of the loss function or after a certain number of iterations. A network may perform poorly because of lack of convergence to validation data (underfitting) or lack of generalizability (overfitting). The choice of loss function is an important part of model design (369), along with the definition of layers and their connection types, which are collectively referred to as network architecture. For example, annotated tumour areas in tissue slices are used as ground truth, and the NN predicts which areas could be classified as tumour in the same slice (370). The loss function estimates how precisely the network predicts tumour areas. After the training phase, the network is applied to predict labels (tumour or healthy) on new tissue slices.

### 3.2 Machine learning or deep learning?

**Figure 3:**
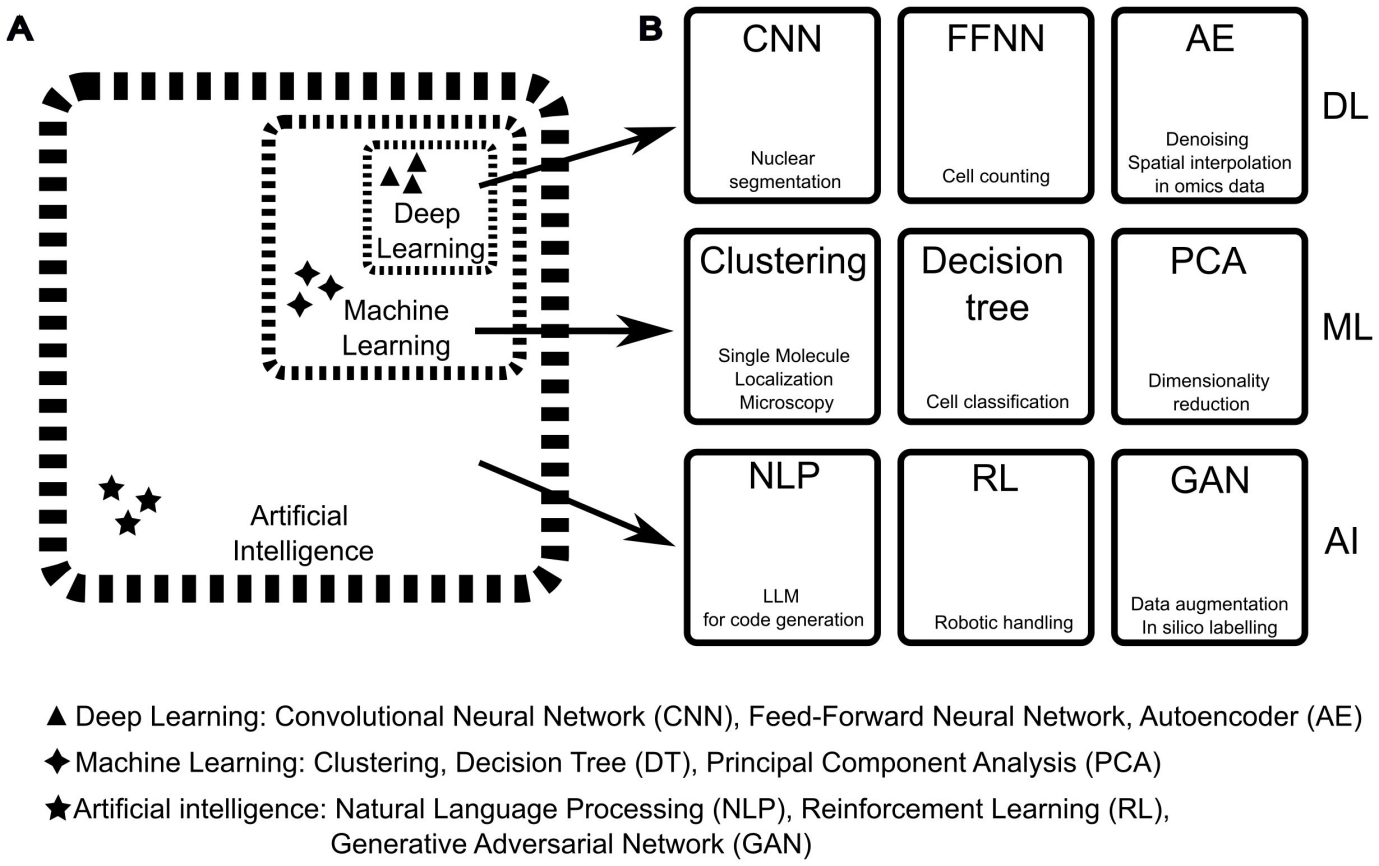
Difference between Artificial Intelligence, Machine Learning, and Deep Learning and examples of methods applicable to microscopy and image analysis. **(A)** The field of Artificial Intelligence **(AI)** builds on several disciplines, such as mathematics, physics, biology, and electronics. AI includes a vast collection of computational methods and includes methods categorised as Machine Learning (ML). The latter includes a subcategory defined as Deep Learning (DL). The boundaries between AI, ML, and DL categories should be considered quite permeable as techniques are shared and new hybrid methods are developed. **(B)** Examples of DL, ML, and AI methods (text on top of the squares) with application to immunological imaging (text on the bottom of the squares). in this figure, the same acronym is used for the singular and plural names of each method. From left to right, for DL: Convolutional Neural Networks (CNN) such as StarDist (371) can be used for crowded nuclei segmentation, Feed-Forward Neural Networks (FFNN) that have been adopted for cell counting (372), Autoencoder networks (AE) that have been used for several analytical tasks such as denoising and spatial interpolation in spatial omics data (373). For ML, from left to right: Clustering methods such as DBSCAN for Single Molecule Localisation Microscopy, Decision Tree methods like random forest for cell classification (322), Principal Components Analysis (PCA) for dimensionality reduction in data analysis including multiple cell measurements (374). For AI, from left to right: Natural Language Processing (NLP) for data mining and code generation (375), Reinforcement Learning (RL) for autonomous improvement of AI models and hardware control (376), and Generative Adversarial Networks (GAN) used for data augmentation to improve the efficiency of image segmentation and interpolation of imaging data sets (377).

Machine learning (ML) is a subfield of AI (**Figure 3A**) that enables systems to learn from data without being explicitly programmed. It focuses on building models or algorithms that can make predictions or decisions by identifying patterns in the data and using them to improve performance over time (378). There are four main types of ML approaches: supervised, unsupervised, self-supervised, and reinforcement learning. Supervised learning is trained on a previously labelled data set that represents *bona fide* the desired outcome, so both the input and desired output are known. This highlights the importance of preparing a training data set that is most representative of the desired outcome, a simple task in principle but one that should be performed with great care (379). By contrast, unsupervised learning finds a structure from the data itself without any prior labelling information. This is a commonly employed technique when unbiasedly clustering information (**Figure 3B**: middle row) and grouping differences in classes. For example, k-means clustering or Principal Component Analysis (PCA, **Figure 3B**: middle row) belong to this category. Self-supervised learning finds a classification by predicting or completing parts of its input, creating labels automatically from unlabelled data. For example, a self-supervised model has been developed to automatically learn semantic relationships between genomic data and improve tasks such as gene annotation or the role of polymorphisms (380). Lastly, Reinforcement Learning (RL, **Figure 3B**: bottom row) involves an agent interacting with an environment and learning through trial and error. It’s often used in broad AI tasks, such as AI-assisted game-playing and autonomous driving systems (376). Any ML workflow comprises a set of input data, a model architecture, and one or more loss functions. ML models can range from low-complexity models with few layers of data transformation – shallow learning – to higher complexity models involving many layers and many connections between them – deep learning.

Deep learning (DL) is a subfield of machine learning (**Figure 3A**) where many layers of data representation are connected to create a complex model with multiple levels of abstraction. Even though many foundational concepts and algorithms have been developed in the twentieth century, practical advancements in DL are relatively recent. Three practical steps have contributed to these advancements and to a general renaissance in the field of AI (368). First, the development of small yet significant algorithms enhanced how these deep stacks of layers can be interconnected (366, 367). Second, bigger storage was available to host the growing training data sets. Lastly, cheaper hardware increased computational power. In fact, DL models are based on simple additions and multiplications of big multi-dimensional arrays of data (called “tensors” in mathematics), and these operations can be easily parallelised. The development of powerful graphical units (GPUs), originally designed to improve the gaming experience but with the ability to be programmed for massively parallelised calculations and scientific computing, led to the implementation of GPU-based NNs (381, 382). Today, along with gaming GPUs, researchers can also leverage dedicated GPUs, optimised for DL tasks, and specialised hardware such as tensor processing units (TPUs), with the potential for requiring less computational resources and becoming an integral part of all domains of science, including microscopy. However, practical implementation of these networks still requires programming skills, with Python being the primary development language; popular frameworks include Tensorflow/Keras (368) and Pytorch (383). DL algorithms are affected by hardware bottlenecks in steps that are not hardware accelerated, therefore some attempts have been made to leverage alternative electronics boards like field-programmable gate array (FPGA, 384), which offer the flexibility of reconfigurable circuitry. For applications requiring low power consumption, FPGA have shown to be from 3 to 5 times more efficient than GPU per processed image, for tasks such as image compression (385). The advent of “liquid” and more efficient hardware such as FPGA will dictate the pace by which AI methods are implemented in microscopy and other fields.

To conclude, ML and DL techniques include computational systems capable of learning from data. Shallow ML systems continue to be utilised for their rapid training, minimal computational resource requirements, and low complexity, allowing for a complete understanding of how they operate. In contrast, DL systems require significantly more computing resources but have considerably higher capabilities, thus aiding all areas of microscopy, from experimental design to image acquisition, analysis, and data mining (386). These two types of systems are often used together in algorithms mixing classic programming, traditional ML, and DL according to the task (this permeability is reflected in the dashed lines of **Figure 3A**).

### 3.3 Image-based machine learning

Image-based ML methods can preprocess images or segment, detect, and track objects within multidimensional microscopy images. The most used approach is supervised learning, which employs a manually segmented data set to train the model. Traditional, shallow ML techniques for image segmentation include Support Vector Machines (SVM) and Decision Tree classification (**Figure 3B**: middle row). Such ML models can yield good results across various applications and are featured in numerous open-source image analysis platforms (ilastik (322), Fiji/ImageJ with WEKA (387) and LABKIT plugins (388), QuPath (332), CellProfiler (323), MIB (389). They are user-friendly, can be used even without programming experience, and demand fewer computational resources compared to DL (388).

On the other hand, DL approaches for image processing have expanded the range of problems that can be solved (366). In the case of image-based methods, they are primarily employing a type of NN called Convolutional Neural Network (CNN, **Figure 3B**: top row, 359, 379). CNNs use layers to extract hierarchical representations of input data, in a way similar to how we learn information on an object by viewing it from different distances or angles. These layers implement two data processing functions: the convolutional filter (hence the name) and the max pooling function. Convolutional filters act like a magnifying glass that scans an input image while applying different kernels (a small matrix) to the image at each position. These kernels help identify specific features within the image, such as edges or corners. The result of this operation is referred to as a feature map, which highlights where these features exist in the image. Max Pool operations are used after convolutional layers to reduce the spatial size of the data while retaining important information, thereby making the network more efficient for computational analysis. Here, we will survey the landscape of current applications of image-based DL methods to the microscopy modalities that we previously discussed, highlighting, where existing, their applications to immunology or virology (**Table 1**).

A major theme in DL applications to many microscopy techniques has been finding ways to increase the wealth of extracted information and overcoming the limitations of the specific techniques, such as increasing resolution without sacrificing acquisition speed. In cryo-ET, DL has been used to learn structural information from single-particle cryo-ET analysis (390) or to achieve isotropic resolution without the need for sub-tomogram averaging (391). In single-particle cryo-EM, DL aided particle model-building by creation of intermediate-resolution maps (392) or model building automation (393). In virology, DL with single-particle cryo-EM has been instrumental to the characterization of tegument architecture in human cytomegalovirus (394). In super-resolution, and specifically in localisation microscopy, DL has been used to increase the acquisition speed, by reducing the number of images needed to reconstruct the structures of interest (109), or to help in localising multiple adjacent emitters in 3D, thus improving volumetric reconstructions (110). In immunology, DL with STED has been applied to identify Zika virus reorganization of the endoplasmic reticulum (111). In SIM microscopy, DL can increase resolution and speed (395–397), thus better capturing live-cell events with lower phototoxicity. In confocal and spinning-disk microscopy, DL can increase image resolution (113, 117), reduce optical aberrations (114), and improve FLIM lifetime determination with low photon budget, as in fast live-cell imaging (115). In TIRF, DL helped in improving single molecule FRET (smFRET) by analysing single molecule traces (112). In wide-field microscopy, DL was used to enhance the resolution and optical sectioning capabilities (30, 123, 124), yielding confocal resolution while improving speed. In intravital, microscopy, DL approaches combined with two-photon excitation and adaptive-optics aberration correction have improved subcellular resolution without sacrificing acquisition speed (133). Finally, DL has been applied to light-sheet microscopy for physics-informed deconvolution, i.e. a combination of DL with optical information on the microscope setup (134). In light-sheet and confocal microscopy, DL also provided axial resolution enhancement, by learning from unpaired high-resolution, 2D confocal images and low-resolution 2D images from other planes (113).

Methods to improve resolution, signal or speed often apply to a specific imaging modality and do not translate well to other image modalities. Recent DL models have tried to provide a more general approach, for example when restoring fluorescence images from all imaging modalities (398), improving resolution without additional data acquisition (399), interpolating images between frames (400) or when performing object detection (401).

Major limitations of fluorescence microscopy are photobleaching, phototoxicity and limited speed when acquiring multiple channels. DL methods can overcome these limitations by providing *in-silico* labelling of transmitted light images (126–129). For example, DL with *in-silico* labelling of brightfield images has been used to improve the tracking in chemotaxis experiments (131), or to predict the lineage choice of differentiating hematopoietic progenitors (130). Finally, DL has been applied to phase, label-free imaging (125), or to achieve fast, volumetric live-cell microscopy of bioluminescent probes (402). In immunology, DL with optical diffraction tomography has been instrumental for label-free tracking of immunological synapse of CAR-T cells (403).

DL methods are now widely used for preprocessing, segmentation, detection and tracking tasks (379, 404). In image preprocessing, DL models are extensively used to denoise fluorescence images, as indicated by the many examples in the literature (290, 398, 405–414). In immunology, DL denoising was instrumental to improve contrast in Imaging Mass Cytometry, thus helping in characterizing the phenotype of immune populations in human bone marrow samples (415). DL helped in separating channels for filter-free imaging (125, 416, 417), and to assist tracking by improving linking accuracy (316, 320, 418). Also, DL is aiding cell phenotyping from multiplex immunohistochemistry images, for example to characterize tumour microenvironment in lung cancer (121) or pancreatic ductal adenocarcinoma (122). Finally, DL models can assess the quality of fluorescence images and identify artefacts (419). Moreover, DL has been applied to denoise low-dose cryo-TEM images (420).

DL is employed in supervised cell segmentation, such as in the case of general models U-Net (421), StarDist (371), Cellpose (422) and Segment-Anything Model (SAM) models (423, 424), which are available as plugins in many open-source and proprietary image analysis software. Specialised models tackle intracellular organelle segmentation (327, 425), segmentation of extracellular vesicles in TEM (103), HIV-1 virions in TEM (104) or mitochondria in FIB-SEM (426). In immunology and virology, DL has been applied to FIB-SEM images of SARS-CoV2 patient-derived platelets to segment α-granules or mitochondria (105). Ligh-sheet microscopy of lungs together with DL-based analysis allowed the spatial profiling of nanoparticle delivery to alveolar macrophages (135). Image segmentation is a challenging part of image analysis in multiplex imaging, where cells are often densely packed. In this case, DL was employed to perform cell segmentation, thus improving single-cell feature extraction (118, 119), or to perform a detection-based classification, therefore providing phenotypic analysis without segmentation (120).

In detection tasks from fluorescence images, DL helped in recognizing apoptotic cells from intravital multiphoton movies (427), phototoxicity in widefield time-lapse experiments (428) or, together with widefield high-content screening, to detect virus-infected cells and predict if they will follow a lytic or non-lytic infection (429). Furthermore, DL with confocal microscopy helped in classifying expression of TLRs from PBMCs of HIV-positive patients under ART therapy (430). In cryo-ET, CNNs are helping annotation and feature extraction for *in situ* identification of structures of the molecular components of interest (431), or template matching, i.e. detection of objects with an arbitrary shape, which is the most widely used approach in cryo-ET for particle picking (106). Still in cryo-ET, DL has been applied for finding macromolecules in cellular 3D tomograms (28). DL detection algorithms can also support feedback microscopy in real time (432) by automatically detecting events to guide acquisition, generate feedback, and predict cell fate.

In tracking, CNNs with intravital multiphoton microscopy have been used to accurately measure the position and shape of CD4+ T cells interacting with plasmacytoid dendritic cells *in vivo*, aiming to study interaction differences in lupus nephritis (132) or to link cell tracks in intravital imaging of leukocytes (433).

One of the limitations of supervised learning is the generation of accurate training data sets, which is, in most cases, a manual task that can be time-consuming and still prone to bias. A possible approach to overcoming this limitation is the use of self-supervised methods. For example, information from the OpenCell database was used to cluster proteins into organelles and individual protein complexes (434). Similarly, in another study DL was used to segment mitochondria, based on a training data set that was generated with DL (435). Such simulation-supervised approach could be, in principle, generalisable to other organelles or even to cellular segmentation in tissues. Self-supervised or weakly supervised models are also employed for cancer prognosis and diagnosis in pathology slides (436).

Overall, image-based DL methods are assisting a wide range of microscopy techniques in tasks from acquisition to image analysis and data extraction (**Table 2**). Many of the above examples are widely applicable to different types of samples, including those related to immunology or virology.

**Table 2:** Examples of AI approaches for selected tasks.

Task	Examples of AI approaches
User-friendly segmentation of cells or organelles	Traditional ML techniques (322, 332, 387–389) or DL plugins (371, 422, 423) within common image analysis platforms
*In-silico* labelling of transmitted light images	(126–129, 131)
Cell segmentation in multiplex imaging	Segmentation-based approaches (118, 437) or segmentation-free phenotyping (120)
Resolution enhancement	In confocal (113) or super-resolution localisation microscopy (109, 110), and SIM microscopy (299, 395–397, 411)
Increasing acquisition speed	DL to reduce the number of acquired planes (202, 438) or provide virtual optical sectioning from widefield microscopy (30, 123, 124)
Detection of phototoxicity in live-cell imaging experiments	Detection of apoptotic cells (427), evaluation of phototoxicity (428)
Denoising of fluorescent images	(290, 398, 405–414)
Improving linking accuracy in tracking	(316, 320, 418)
Clustering of phenotypes or behaviours of immune cells	Traditional ML for phenotyping multiplex images (206, 439). DL for clustering phenotypes in high-content screening (374). DL to cluster behaviours from intravital cell dynamics (440)
Integrating microscopy data with multi-omics	(29, 441, 442)
Dialoguing with imaging data and software	(443)

The table summarises the main approaches employing traditional ML or DL for selected tasks in image analysis and immune phenotyping.

### 3.4 Data-based machine learning

Data-based ML can analyse, interpret, and learn from data. In this Review, we refer to data-based methods as the ones that can be applied generally to numerical or categorical data without the specific need for data to be generated with imaging techniques. For example, data-based ML is used in microscopy when clustering data extracted from images with the previously described image-based methods (442, 444), or when combining information from microscopy with text data generated with other methodologies, such as genomics or proteomics data (445). These tasks can be approached using traditional, shallow ML or DL.

Techniques using traditional ML include linear regression, logistic regression, and decision trees, such as Random Forest. For example, logistic regression was employed to predict MHC ligand, where a binding model and an antigen processing model were combined, and results were classified according to logistic regression score (446). Random forest was applied to analyse T cell-dendritic cell interaction in a lupus nephritis model (132).

Instead, unsupervised learning techniques are employed when the desired outcome is unknown or input data are not labelled. In this case, unsupervised learning can help clustering data in groups. Notable examples are the K-means, DBSCAN, and an unsupervised version of random forest algorithms (378). Clustering techniques have been used in high-throughput screenings to highlight differences between biological conditions when segmenting and measuring cells (374), or, in immunology, to cluster signatures and perform neighbourhood analysis in multiplex imaging in tissues (206, 439, 447) and single cells (246).

Another set of techniques, called dimensionality reduction, is used to group variables into “super variables”. Techniques falling in this category are PCA, t-SNE, and UMAP (378). Dimensionality reduction has been used to classify cell cycle and disease progression after feature extraction with CNNs (448), to segment touching cells in confocal and two-photon microscopy (449), to group clonal distribution of CD4+ T cells in gut epithelium following *Listeria monocytogenes* infection (450), or to obtain behavioural signatures of immune cells in intravital inflammation models, guiding the discrimination between pathogenic and non-pathogenic phenotypes (440). Also, dimensionality reduction techniques are essential in multiplex imaging when grouping cell phenotypes (240, 451).

DL for data-based methods can employ different types of architecture depending on the purpose. An Autoencoder Network (AE) is a type of NN that learns to encode input data into a lower-dimensional representation and then decode it back into the original form, thus learning meaningful representations from the data. This can be helpful for tasks like dimensionality reduction or feature extraction. AE (**Figure 3B**: top row) has been used to combine low-dimensional representations of scRNA data, generated using large language models, with actual single-cell scRNA-seq data from different species to create a supergene classification that can bridge differences between individual single-cell experiments and different species (452), or to create deep generative models for spatial-omics analysis that can take into account the spatial relationship information (373). A Feed-forward neural network (FFNN, **Figure 3B**: top row) is another type of NN where information flows only in one direction (forward) through layers of interconnected nodes. FFNNs are commonly applied to regression and classification tasks, as they can learn complex non-linear relationships between inputs and outputs. For example, a feedforward network was used to classify cell tracks in 3D biomimetic gels of immune cells co-cultured with breast cancer cells in organ-on-chip (453).

Recurrent Neural Networks (RNNs) excel at processing sequential data like natural language text, where the context of previous words influences the use of subsequent ones. After dividing data into small chunks – called tokens –, RNNs process them recursively to generate the most likely information based on the previous information. For example, this type of network could predict the cell lineage of hematopoietic cells from brightfield images by extracting time signatures of cells from image features extracted with a CNN (130).

On the other hand, transformer networks weigh the importance of input tokens to construct a connection map without requiring sequential processing (454). They are at the basis of the Natural Language Processing (NLP, **Figure 3B**: bottom row) chatbots used today, such as ChatGPT, and are also called Large Language Models (LLMs). These tools can serve as a valuable resource aiding researchers in designing microscopy experiments (455) or drafting algorithms to implement the ML techniques described here. LLMs can be applied to data mining in scientific data sets, such as those generated from single-cell omics (as reviewed in 456), and some attempts exist to apply it to bioimage analysis (443, 457). We also foresee that these techniques will be increasingly integrated in microscopy hardware, for AI-assisted sample exploration and acquisition.

Another type of generative network is called Generative Adversarial Network (GAN, **Figure 3B**: bottom row). This network comprises two networks, one generating new data and the other evaluating its suitability as output. The generating network challenges the evaluating network, thus introducing an element of randomness and “creativity” in the output (458). This broad class of networks can be used to generate models of protein structures (the general AlphaFold (459) and its open version OpenFold (460), or a more specific version for proteins of the immune system (461)) or to reconstruct molecules from cryo-ET tomograms (390, 462). Generative neural networks informed on the features of highly metastatic melanoma by “reverse engineering” a supervised CNN for cell classification. In this example, a CNN is initially trained on patient-derived melanoma xenografts to classify them based on their metastatic capability. Then, a generative neural network is used to create *in silico* cell images with exaggerated features, which are then used to analyse which features in the CNN are most prevalent (463). This approach is particularly intriguing as it uncovers new quantitative insights within the hidden features of DL, thereby providing information that could potentially lead to the generation of new scientific hypotheses.

In summary, we outlined some applications of data-based ML techniques for microscopy (**Table 2**). The field is vibrant and complex, and evolving at a fast pace. Shallow ML and DL can be employed to cluster information and combine microscopy data with multi-omics data (29, 441, 442), or to predict molecular biomarkers from pathology images (464). These technologies could aid vaccine design, as outlined in Hederman and Ackerman (465) or to improve antibody design (466). As such, a dialogue between computer scientists, microscopists and immunologists is fundamental.

### 3.5 Impact of open-source software in AI deployment and democratization

AI can hugely facilitate the analysis of large image data sets and the characterisation of rare biological events. Because OSS solutions can rely on the contribution and critical judgement of the wide imaging community, they are indispensable for the development of trustable AI applications for bioimage analysis. In this regard, several software tools have been deployed during the past years, both as plugins for the major software GUI such as FIJI (StarDist (371), Cellpose (467), DeepImageJ (468)) or napari (SAM (423)), or as code notebooks (ZeroCostDL4Microscopy (335)).

In the previous sections, we outlined the main DL applications to analyse bioimage data, many of which are distributed as open-source software. Although some of these applications were not specifically developed with immunology in mind, their usage can significantly benefit immunological imaging. For instance, existing code can be adapted for specific biological questions, or their training data can be used to enhance other domain-specific DL models.

Furthermore, publicly available image databases are key in truly open-source AI models (469), as outlined by the Open-Source AI Definition^9^. In this sense, data sets specific to the immunological field (319), or for broader imaging purposes, such as the ones hosted on BioImage Archive (470), can constitute a valuable resource of image data to test AI software tools and foster immunological research. These data sets should follow the “Findable, Accessible, Interoperable, Reusable” (FAIR) principles (471).

Finally, the use of LLMs or other AI methods to aid software creation (443) will constitute an essential part of AI deployment and democratisation, as it empowers every scientist, even without programming experience, with the “wisdom of the crowd” provided by AI training data sets that can summarise a vast amount of human knowledge.

### 3.6 AI for instrument control

The role of AI for instrument control and automation can be delineated in at least two different ways: the first being the support of AI in automating the development of open-source code (443) for feedback microscopy, while the second involves utilising the AI-based methods described above to interpret the image data, thereby revealing information that can be used to redirect the acquisition process.

The use of open-source platforms Micro-Manager (349) or Pycro-Manager (348) for microscope control can be integrated with Python packages such as Scikit-Image^10^ (472) for image segmentation and Scikit-Learn^11^ (473) to run ML tasks and feed results back into the acquisition software. As a practical example, DL segmentation methods have been used to identify cells and set the correct acquisition parameters (474) or switch microscopy modality (31) to image immunological synapses. Furthermore, cost-effective open hardware facilitates possible integrations of the acquisition microscope with AI feedback tools, for example to control anaesthesia, temperature, and humidity in intravital imaging (similarly to approaches tested in the clinics, 475, 476), or correcting the state of the optical system by acting on adaptive optics to minimise the loss of signal (238).

The implementation of AI for instrument control is automating the execution of precise and complex hardware tasks, shifting the troublesome duties from the human to the machine. In our view, rather than totally delegating the control of the experiment and the handling of expensive instruments to the automatic agents (as a kind of hardware/software AI-equipped decision maker), the implementation of AI tools should work as advanced technology to help the human researcher in steering the course of the experiment. In this regard, the integration of large language models and feedback microscopy is foreseen as the future evolution of microscopy, where the user doesn’t necessarily need to be highly skilled in all the aspects of microscope acquisition, hardware control, bioimage analysis: a microscopy platform could accept human language instructions and convert these into hardware control operations to provide the requested type of data sets (477).

## 4 Discussion

In this Review, we surveyed the primary applications of light and electron microscopy in immunology and virology for preclinical research, outlined the concepts of AI, ML, and DL, and explored their current uses in microscopy, image analysis, data analysis, and feedback microscopy. Although we mentioned studies employing microscopy and AI in immunology, the intersection of these fields remains largely unexplored. For instance, many of the tools discussed are designed for specific applications, and few attempt to integrate multiple imaging modalities or tasks. Lack of generalizability, when not due to training issues, is a major challenge in the current research on DL and microscopy. On this topic, Kawaguchi et al. showed that analytical insights into building more generalizable architectures could be drawn when using specific loss functions and concluded on the importance of human reasoning on the physical properties and engineering principles of the specific problem at hand (478). We believe this capability could arise either through AI-assisted multimodal visualisation or through a combination of direct visualisation and AI computational modelling of structures or dynamic events.

DL networks concatenate many hidden layers to generate a rich output, and although these layers are just composed of numbers and simple operations, understanding how the output relates with their inner functioning is exceptionally challenging. Also, the mathematical structure of DL makes it prone to hallucinations, i.e. the generation of plausible but incorrect output (479). These two problems could be seen as epistemically relevant if we treated the output as scientific knowledge in its own right, without further experimental verification (480). Rather than seeing this as a problem, we think that integrating DL with additional local data, for example from microscopy or other techniques, could enhance our ability to interrogate data and generate additional perspectives (27, 481), potentially inspiring new scientific ideas, to be later supported by rigorous verification, or highlighting the limitations of current theories (482). Finally, the use of AI in real-time contexts requires an evaluation of the possible outcomes of AI hallucinations, with respect to model reliability and consistency. In this regard, the openness of the AI-decision making process, the integration with traditional techniques and a dialogue with the human researcher still have a prominent role.

While one might be tempted to attribute some level of understanding to AI models, it is essential to recognise that these models merely process numerical representations of data generated through mathematical transformations (368). As such, their ability to think is as limited as the size of the data set and the types of possible transformations. This highlights the importance of dataset preparation to achieve reliable outcomes, as well as the need of a community effort for more curated, freely accessible, “FAIR” (471) microscopy data. The careful evaluation of possible cognitive biases during curation of training datasets is very important (483). For example, researchers can assess the imbalance between classes, i.e. the under-representation of certain types of conditions during data generation, the uniformity of data acquisition across instruments or laboratories, the quality of annotations or the methods used for data augmentation (484). Also, the application of AI might require considerations about data privacy and the openness of AI models, especially when relying on external services for the AI processing. Steps to make AI more accessible and broaden its usage include creating efficient models that require less hardware resources and could be used on low-cost computers, or designing better user interfaces to guide the user in all the phases of AI implementation, such as with data quality assessment and data preparation, with the choice of NN architecture, with model validation and during model usage. For example, projects like DeepImageJ (468) and its model sharing platform BioImage Model Zoo^12^ are going in this direction. Finally, a rigorous determination of the amount of scientific data needed to obtain reliable training is required, as well as techniques for performing automatic choice of DL architecture and training (485).

A cross-disciplinary approach that includes skills in biology, microscopy, electronics, and software programming is necessary for implementing AI-based hardware and software tools. This approach could help shaping open, local models with efficient use of hardware resources, to the benefit of real-time hardware control. To achieve this fully integrated use of AI tools, LLMs can help in data mining and code drafting tasks but are still limited in their capabilities to precisely manipulate factual knowledge, for example to provide advice on microscopy. A step towards more specific and fact-based AI tools is represented by Retrieval-Augmented Generation (RAG) models (486). Ideally a general model, for instance a chatbot such as BioImage.IO (455), could understand better the initial request for help posed by a human researcher (e.g. “find the organelle in these cells”) and could drive a more specialised model, for example a CNN, to perform specific tasks (e.g. segmenting specifically mitochondria).

Looking ahead, we can imagine a future where multiple AI models, or agents, could act on specific parts of the microscopy process (487). New DL agents could integrate fact-checked advice on experiment design with code generation models, AI-based hardware control and analysis models, to assist the user across the whole research cycle. Integrating AI, imaging data science and microscopy automation will allow the automatic monitoring of the sample, the detection of anomalies, and the adaptive change of hardware to guide the acquisition of key biological events, thus enhancing our visualisation capabilities across scales and our systemic understanding of the immune system.

## Footnotes

1
https://www.rstudio.com

2
https://github.com/micro-manager/pymmcore

3
https://www.arduino.cc

4
https://www.raspberrypi.com

5
https://librehub.github.io

6
https://www.glencoesoftware.com

7
https://www.zeiss.co.uk/microscopy/home.html

8
https://abberior.rocks/superresolution-confocal-systems/imspector

9
https://opensource.org/ai

10
https://scikit-image.org

11
https://scikit-learn.org

12
https://bioimage.io
